# Supporting healthcare professionals in a remote rural area of Tanzania

**DOI:** 10.1080/17571472.2018.1453967

**Published:** 2018-03-27

**Authors:** Sylvia Berney, Helen Halpern

**Affiliations:** a Barnet and Royal Free Hospital, London, UK; b Independent Scholar, London, UK

**Keywords:** Professional development, healthcare professionals, professional support, health in developing countries

## Abstract

In October 2017, as two semi-retired NHS doctors, we visited a local hospital and a health centre in Sumbawanga, a rural town in Tanzania. We were curious to find out how the healthcare system worked, to see what support might be helpful to the healthcare professionals themselves and whether and how ideas might be applied in the U.K. We found the health facilities we visited to be well organised and functioning with a small number of multi-skilled clinicians. However, we were aware that there are inadequate numbers of suitably trained healthcare professionals per head of the population in this area and that this could contribute to some of the poorer health outcomes. Our visit left us wondering whether the provision of support in the form of leadership coaching, educational consultancy and friendship with colleagues in the U.K. might enhance job satisfaction and, in turn, whether this might have a beneficial effect on staff recruitment and retention. These are ideas that we are now pursuing with a plan to return to Tanzania in the autumn of 2018.

## Why this matters to us

After more than 30 years of working in the NHS in London, we wanted to explore ways that our experience of clinical medicine, medical education and professional development might be useful to healthcare in Tanzania and also to identify things the NHS could learn from Tanzania. We had both enjoyed visiting Tanzania as tourists and decided to return in a different capacity. We wanted to share ideas about the delivery of healthcare and the provision of support for healthcare professionals, possibly with a view to a longer term relationship. As we had a connection with a rural town through a friend who runs an education project there, this was where we decided to start. In October 2017, we visited Sumbawanga in the province of Rukwa with the aim of contacting healthcare practitioners to find out about their experience of living and working there.

## Key messages

Health care professionals working in a rural area of Tanzania are multi-skilled and dedicated. However, there are insufficient numbers relative to the needs of the local population. There may be mutually beneficial opportunities for support and learning between healthcare colleagues in Tanzania and the UK.

## Background

The United Republic of Tanzania is situated in East Africa. The mainland, Tanganyika, had been a United Kingdom–United Nations trust territory and gained full independence in 1961, becoming a republic in 1962. The island of Zanzibar gained independence in 1963 and merged with Tanganyika in 1964 to form the United Republic of Tanzania.

At 945,087 km^2^, Tanzania is almost 4 times the size of the U.K. but its population is only 43.5 million compared to that of the U.K. (55 million). It has a well-established healthcare infrastructure and the Ministry of Health states that it is ‘committed to facilitate the provision of basic health services that are good, quality, equitable, accessible, affordable, sustainable and gender sensitive’.

In Tanzania, there is a particularly great need for healthcare provision in obstetrics, contraception and paediatrics, as 49.3% of the population is aged under 15. In addition, the maternal, infant and under-five mortality rates are high, although comparable to other developing world countries (Table 1).

**Table 1.** Comparison of mortality rates (according to World Bank data).

	Tanzania	Kenya	Zambia	U.K.
Maternal mortality ratio modelled estimate per 100,000 live births (2015)	398	510	224	9
Infant mortality per 1000 live births (2016)	40	36	44	4
Under five mortality per 1000 live births (2016)	57	49	63	4

There are 31 regions in Tanzania. At 22,792 km², the region of Rukwa is the same size as Wales. However, its population of just over 1 million is about a third of that of Wales.

As Tanzania has a predominantly rural population, with 70% of people living in the countryside, the delivery of healthcare is challenging. Rural areas, such as Rukwa, are particularly disadvantaged in comparison to urban ones in lacking trained healthcare professionals. As a World Health Organization case study on Tanzania states [1], ‘health issues of women are not adequately addressed to cover their needs. Furthermore, health system referral is weak. Although regional referral hospitals are in place in all regions, they are challenged by insufficient availability of key clinical staff’. Table 2 shows the numbers of different health care professionals (cadres) in each region of Tanzania and this illustrates how Rukwa is one of the more disadvantaged regions.

**Table 2.**
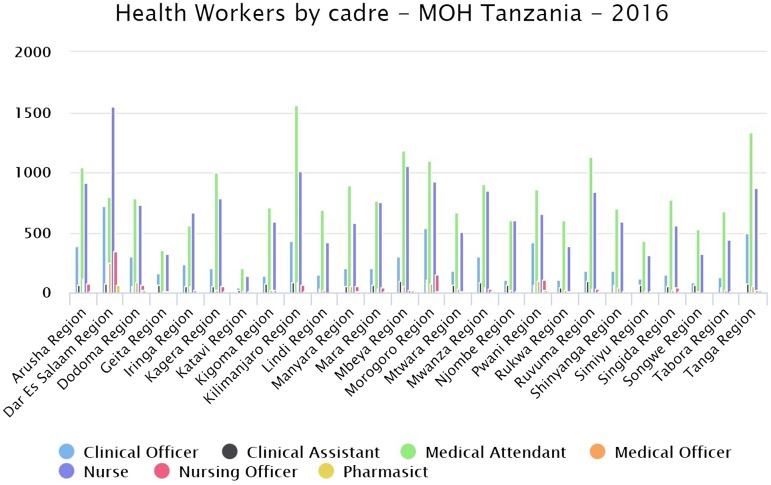
Healthcare workers by cadre in regions of Tanzania (figures are from the Tanzania Ministry of Health Data Portal).

Shortages of health workers are a critical public health issue in many countries [2]. As one of the more remote, rural areas in Tanzania, Rukwa region has problems attracting and retaining healthcare professionals. This is partly because of its distance from big cities and universities. It is a 1 h flight from Dar es Salaam to Mbeya and then a dusty, 6 h bus journey to reach Sumbawanga. Another possible reason is ‘due to poor working and living environments’ [3]. In order to attempt to mitigate this, the government has increased salaries and built houses to encourage health care workers to the area [4].

The health professionals and senior administrators to whom we spoke reported that they were working there because they had local family. We wondered whether identification with their local community could be an important motivator for working in the area. However, we were concerned that they could be missing out on systems of professional development and support that might be available if they were based somewhere with more developed healthcare facilities and a wider network of healthcare professionals.

Sumbawanga is the regional capital of Rukwa. It is a small town with a population of just under 210,000 people living in the municipality. It has the one regional government hospital, and also a private hospital and health centre both run by the Catholic diocese. Dispensaries and pharmacies supplement the provision of healthcare in the town and rural villages. In addition, many people consult traditional healers.

**Photo 1.**
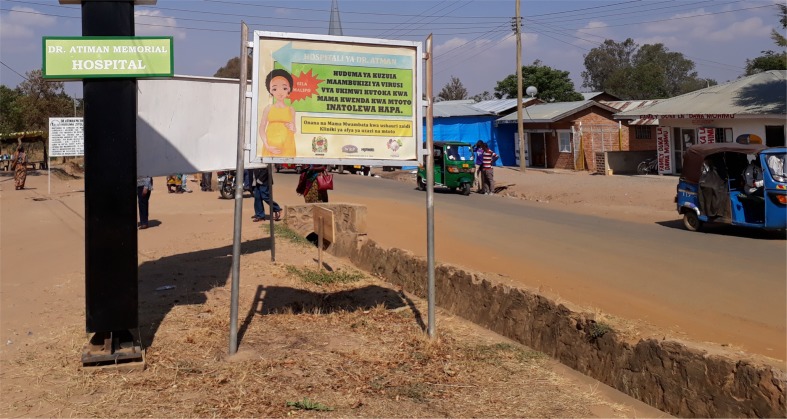
To show the street outside the Dr. Atiman Memorial Hospital.

We had a letter of invitation from the local bishop and so were able to visit the Dr. Atiman Memorial Hospital and the health centre in Sumbawanga, both run by the Catholic diocese. However, as we did not have the requisite Ministry of Health approval, we could not visit any government health facilities (Photo 1).

We met the senior administrators and doctors and saw the breadth of healthcare provision in the Dr. Atiman Hospital and health centre. We were impressed by these professionals who are multi-skilled and achieve a lot with few resources. For example, a doctor trained as a dermatologist was also able to perform Caesarean sections; and low birth weight (under 2.5 kg) neonates were cared for using skin-to-skin ‘kangaroo mother’ care as there are no incubators.

**Photo 2.**
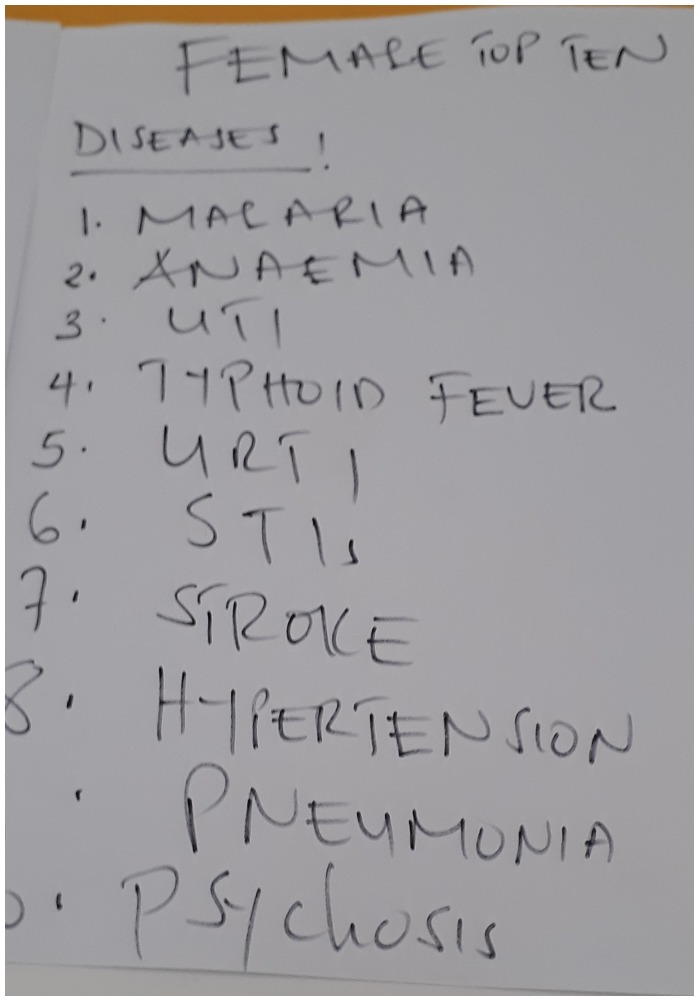
Poster of ‘Top Ten’ conditions seen on female medical ward.

The hospital has six wards, a dedicated outpatient clinic and a dispensary (Table 3). Staff at the Dr. Atiman Memorial Hospital have made posters on the wall of each ward of the ‘Top Ten’ diseases seen by them (Photo 2).

**Table 3.** ‘Top Ten’ conditions seen in the paediatric and female wards.

	Paediatric	Women’s medical	Women’s surgical
1	Pneumonia	Malaria	Malaria in pregnancy
2	Anaemia	Anaemia	Anaemia in pregnancy
3	Malaria	Urinary tract infection	Abortion complications
4	Septicaemia	Typhoid fever	Ectopic pregnancy
5	Malnutrition	Upper respiratory tract infection	Intestinal obstruction
6	Urinary tract infection	Sexually transmitted diseases	Fractures
7	Amoebiasis	Stroke	Gynaecological diseases
8	AIDs	Hypertension	Septic wounds
9	Upper respiratory tract infection	Pneumonia	Osteomyelitis
10	Intestinal worms	Psychosis	Burns

As part of its out-patient services, the hospital has a dedicated HIV clinic and the government provides free drugs for this condition. Interestingly, the current prevalence of HIV in Sumbawanga has risen from 4.9 to 6.2% over the last few years. The increased movement of people associated with the construction of a water pipeline and a tarmac road to Sumbawanga might account for this increase.

While visiting the hospital we met the director of the St Bakhita Training Institute in Namanyere which is two hours from Sumbawanga by road, and is one of 22 health training institutes in the country registered with the National Council for Technical Education. It was established in 2004 to provide health education and to train nurses, clinical medical officers and laboratory technicians to certificate and diploma level for the local area. The hope is that many of these healthcare professionals will stay to work in the region once they have qualified.

We plan to remain in contact with Rukwa medical educators, senior administrators and doctors whose English is sufficiently good to communicate with us as we do not know the national language, Kiswahili. The aim will be to share ideas and good practice and to support each other in the professional challenges faced by doctors and health professionals in both countries. This will take place via WhatsApp conversations to provide a free platform for listening and thinking.

Now back in London, we have initiated conversations with some of the senior healthcare leaders we met and have also made a connection with Tuheda, the Tanzania UK Healthcare Diaspora Association. Some of our ideas for taking things forward include the establishment of further lines of communication, writing case studies and looking out for low tech, community initiatives that could be implemented in either country.

We plan to return to Sumbawanga in autumn 2018 to visit our new colleagues and have applied for permission to make contact with healthcare professionals at the Government Hospital. We also intend to go to St Bakhita Training College to meet students and teachers and to be involved in training there. We are keen to find out how to recruit and retain healthcare professionals in local areas where there is a shortage of key staff. This issue is relevant to a range of professions in high, middle and low income countries. Our aim is to create a network of interested people who will give their time voluntarily and with no financial cost involved in any of the projects. Although we are uncertain where this work may lead, we are open to it evolving in a range of creative directions.

## Disclosure statement

No potential conflict of interest was reported by the authors.
